# Determination of polychlorinated biphenyls in marine fish obtained from tsunami-stricken areas of Japan

**DOI:** 10.1371/journal.pone.0174961

**Published:** 2017-04-04

**Authors:** Yoshinori Uekusa, Satoshi Takatsuki, Tomoaki Tsutsumi, Hiroshi Akiyama, Rieko Matsuda, Reiko Teshima, Akiko Hachisuka, Takahiro Watanabe

**Affiliations:** National Institute of Health Sciences, Setagaya-ku, Tokyo, Japan; National Sun Yat-sen University, TAIWAN

## Abstract

We determined the polychlorinated biphenyl (PCB) congeners in 101 marine fish obtained from tsunami-stricken areas following the Great East Japan Earthquake in 2011. In particular, to determine the degree of PCB contamination in the fish, we investigated the concentration of total PCB (∑PCB) and the proportions of 209 individual PCB congeners by high-resolution gas chromatography/high-resolution mass spectrometry. The ∑PCB concentration was 1.7–33 ng/g in fat greenling (*n* = 29), 0.44–25 ng/g in flounder (*n* = 36), and 1.6–86 ng/g in mackerel (*n* = 36), all values being much lower than the provisional regulatory limit in Japan. In the congener analysis, tetra-, penta-, hexa-, and hepta-chlorinated PCB congeners dominated in all samples (comprising over 86% of the ∑PCB). The proportions of the chlorinated PCB congeners were similar to the contamination patterns derived from Kanechlor in the environment, implying that the marine fish were not contaminated with fresh PCBs.

## Introduction

Following the Great East Japan Earthquake in 2011, the contamination of foods by chemical pollutants, including radioactive materials, heavy metals, and hazardous organic compounds, has roused great concern. Radioactive substances in foods have been controlled by establishing the maximum residue level in food regulations and by monitoring the radioactive cesium concentration in foods [1–3]. However, despite the high likelihood of food contamination by other chemical pollutants after the disaster, investigations of such contaminants have scarcely been reported. If specific hazardous chemicals from industrial or medical facilities damaged by the tsunami have indeed dispersed into the environment, then the health risks incurred by dietary intake of these chemicals need to be managed. Among the candidate hazards are polychlorinated biphenyls (PCBs), which may have leached at high concentrations from old stored electrical condensers and transformers washed away by the tsunami [4].

PCBs have one to ten chlorine atoms attached to a biphenyl and include 209 homologous compounds known as congeners [5, 6]. Being easily soluble in hydrophobic solvents, environmental PCBs tend to accumulate in organisms, particularly in their fatty tissues and are transferred through species along the food chain. PCBs were commercially produced as congener mixtures that achieved desired properties, such as high dielectric constant, high stability, and inflammability. PCBs with various properties were previously used in numerous industrial materials such as paints, hydraulic fluids, and insulating oil for condensers and transformers [4–8]. However, both dioxin-like PCB (DL-PCB, whose chemical structure and steric configuration of dioxin are similar to PCB) and non-DL-PCB (NDL-PCB) are potentially toxic to humans and other organisms [9, 10]. Thus, many countries have banned the use and production of PCB to reduce health risks and environmental loading of these compounds.

Herein, we focused on the PCB contamination in marine fish purchased from market places in the tsunami-stricken area. Fish have been identified as the main source of PCB and dioxin ingestion in the Japanese diet [11, 12]. Thus, consumption of marine fish containing high concentrations of PCB could lead to serious exposure. In this study, to examine whether the tsunami had freshly contaminated marine fish with PCB, we investigated not only the concentration of total PCB (∑PCB) but also the proportions of the 209 PCB congeners in fish purchased from markets in tsunami-stricken areas. The analysis was performed by high-resolution gas chromatography/high-resolution mass spectrometry (HRGC/HRMS). In addition, we aimed to confirm the availability of PCB indicators for estimating the concentrations of residual ∑PCB and total NDL-PCB (∑NDL-PCB) derived from Kanechlor (KC) in Japan.

## Materials and methods

### Samples

We went to general food markets in tsunami-stricken areas (the Aomori, Iwate, Miyagi, and Chiba prefectures) and purchased a total of 101 fish samples, comprising fat greenling (*n* = 29), flounder (*n* = 36), and mackerel (*n* = 36), in 2012–2013. The localities the fish originated from were confirmed by their labeling and by hearing from the seller that these fish were mostly caught near the above areas. Further informational details will be made available upon request. The fish were transported from the markets under refrigeration and were fileted as soon as possible using a kitchen knife to obtain their muscles. The muscle tissue of each fish was uniformly homogenized in a food processor (GM200, Knife mill GRINDOMIX, Verder Scientific Co., Ltd., Germany), and the homogenized fish samples were stored at –20°C until required for analysis.

### Materials and equipment

Dioxin-analysis-grade *n*-hexane, acetone, toluene, dichloromethane, and alumina were obtained from Kanto Chemical Co., Inc. (Japan). A multilayer silica gel column filled (from bottom to top) with 2 g of anhydrous sodium sulfate, 0.9 g of silica gel, 3 g of 44% (w/w) sulfuric acid-impregnated silica gel, 0.9 g of silica gel, and 2 g of anhydrous sodium sulfate was purchased from GL Science Inc. (Japan). PCB standards for calibration (TPCB-CSL-A, CS1-A, CS2-A, CS3-A, CS4-A, and CS5-A) and ^13^C_12_-labeled internal standards (TPCB-CL-A100 and TPCB-SY-A100) were obtained from Kanto Chemical Co., Inc. Retention times were checked by a PCB standard (a mixture of the 209 congeners) prepared by mixing equal amounts of M-1668A-1–0.01x, 2–0.01x, 3–0.01x, 4–0.01x, and 5–0.01x standards (AccuStandard Inc., CT, USA). High-resolution gas chromatography/high-resolution mass spectrometry (HRGC/HRMS) was performed with an HP-6890 Plus gas chromatograph (Hewlette Packard Co., CA, USA) coupled with a JMS-700 MStation mass spectrometer (JEOL, Japan).

### HRGC/HRMS analysis

The extraction and clean-up procedures of PCBs from fish samples are described in S1 Text. All 209 PCB congeners were determined by HRGC/HRMS with an HT8-PCB capillary column (0.25 mm × 60 m, Kanto Chemical Co., Inc.), as previously described [13], with minor modifications. The samples (2 μL) were injected in splitless mode. The measurements were performed in selected ion monitoring mode at a resolution of >10,000 (S1 Table). For each sample whose ^13^C_12_-labeled recovery standard concentration satisfied a 40–120% recovery rate, the PCB concentration was calculated by setting PCB congener concentrations lower than the limit of quantitation (LOQ) equal to zero. The results were analyzed statistically using the Mann–Whitney *U* test and Pearson’s *R* test.

## Results and discussion

### Quality assurance and quality control

First, we evaluated the performance of the developed method. A recovery test was performed on a bigeye tuna sample spiked with 62 representative PCB congeners at concentrations of 0.005 ng/g and 0.05 ng/g. The mean recovery ranges of the 0.005 ng/g and 0.05 ng/g spike levels were 73–115% and 76–113%, respectively (S2 Table). The relative standard deviation was below 10% in the sample of 0.05 ng/g spike level. The limit of detection (LOD) and LOQ were conventionally determined by evaluating the signal-to-noise ratio (S/N) of a five-fold dilution of the lowest standard. The LOD and LOQ for congeners that were not included in the standard were determined by the mean of S/N of the congeners with the same number of chlorine atoms. When the congener was detected in the blank sample, the standard deviation (SD) of the analytical results for the corresponding congener in the five-replicated blank sample was calculated, and the LOD (3 × SD) and LOQ (10 × SD) were determined if these values were higher than the values obtained from the S/N. In this study, the LODs and LOQs of the PCB congeners were 0.035–1.0 pg/g and 0.12–3.4 pg/g, respectively (S3 Table). These results suggest that the developed method can adequately determine trace levels of PCB in fish samples.

### Total PCB (∑PCB) concentration

**Fig 1:**
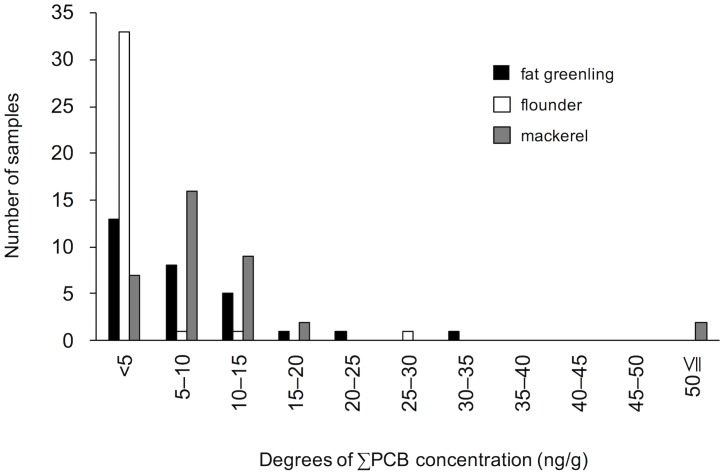
Histograms of the ∑PCB concentrations (ng/g) in fish samples. Bars show fat greenling (black bars, *n* = 29), flounder (white bars, *n* = 36), and mackerel (gray bars, *n* = 36).

PCB was detected in all 101 samples, and the concentrations of 109–198 congeners exceeded the LOQ. A histogram of the ∑PCB concentrations (ng/g) is shown in Fig 1. In approximately 90% of the samples, the ∑PCB concentrations were below 15 ng/g. Most of the fat greenling and flounder samples contained up to 5 ng/g ∑PCB, whereas most of the mackerel samples contained 5–10 ng/g. Very few samples contained high levels of ∑PCB, which was consistent with typical concentration patterns of hazardous chemicals in foods [14]. The maximum concentration obtained from a mackerel sample (Table 1), was 86 ng/g, much lower than the provisional regulatory limit for ocean fish in Japan (500 ng/g) [15].

**Table 1:** Statistical data on each chlorinated congener concentration (ng/g) in fat greenling, flounder, and mackerel.

		Concentration (ng/g)				
Fish group		Mean	SD	Maximum	Median	Minimum
fat greenling (*n* = 29)						
	MoCBs	0.0011	0.0014	0.0061	0.00045	0.00016
	DiCBs	0.026	0.045	0.25	0.016	0.0015
	TrCBs	0.22	0.19	0.68	0.13	0.031
	TeCBs	1.1	0.90	3.5	0.78	0.20
	PeCBs	2.4	2.3	13	1.7	0.53
	HxCBs	2.9	2.4	12	2.1	0.63
	HpCBs	1.2	1.1	4.8	0.75	0.24
	OcCBs	0.17	0.16	0.77	0.098	0.030
	NoCBs	0.017	0.014	0.050	0.011	0.0026
	DeCB	0.013	0.014	0.053	0.0070	0.0026
	∑PCBs^1)^	8.1^*a*^	6.8	33	5.9	1.7
flounder (*n* = 36)						
	MoCBs	0.00056	0.00061	0.0024	0.00028	0.00012
	DiCBs	0.014	0.015	0.075	0.0085	0.00097
	TrCBs	0.15	0.37	2.2	0.050	0.013
	TeCBs	0.52	1.2	6.9	0.20	0.059
	PeCBs	0.85	1.5	8.6	0.40	0.11
	HxCBs	0.85	1.0	5.7	0.52	0.16
	HpCBs	0.33	0.34	1.5	0.21	0.054
	OcCBs	0.048	0.051	0.23	0.028	0.0046
	NoCBs	0.011	0.014	0.062	0.0065	0.0024
	DeCB	0.0097	0.013	0.054	0.0038	0.0011
	∑PCBs^1)^	2.8^*b*^	4.4	25	1.5	0.44
mackerel (*n* = 36)						
	MoCBs	0.0036	0.0037	0.022	0.0031	0.00028
	DiCBs	0.062	0.068	0.43	0.051	0.0015
	TrCBs	0.38	0.52	3.3	0.32	0.026
	TeCBs	1.8	2.7	16	1.3	0.17
	PeCBs	3.7	5.1	27	2.3	0.41
	HxCBs	4.0	5.2	28	2.6	0.60
	HpCBs	1.3	1.6	9.1	0.88	0.19
	OcCBs	0.17	0.23	1.3	0.11	0.020
	NoCBs	0.031	0.024	0.11	0.024	0.0031
	DeCB	0.031	0.018	0.094	0.030	0.0072
	∑PCBs^1)^	11^*a*^	15	86	7.8	1.6

^1)^ Significantly different (*p* = 0.05) results are distinguished by indices *a* and *b*.

**Fig 2:**
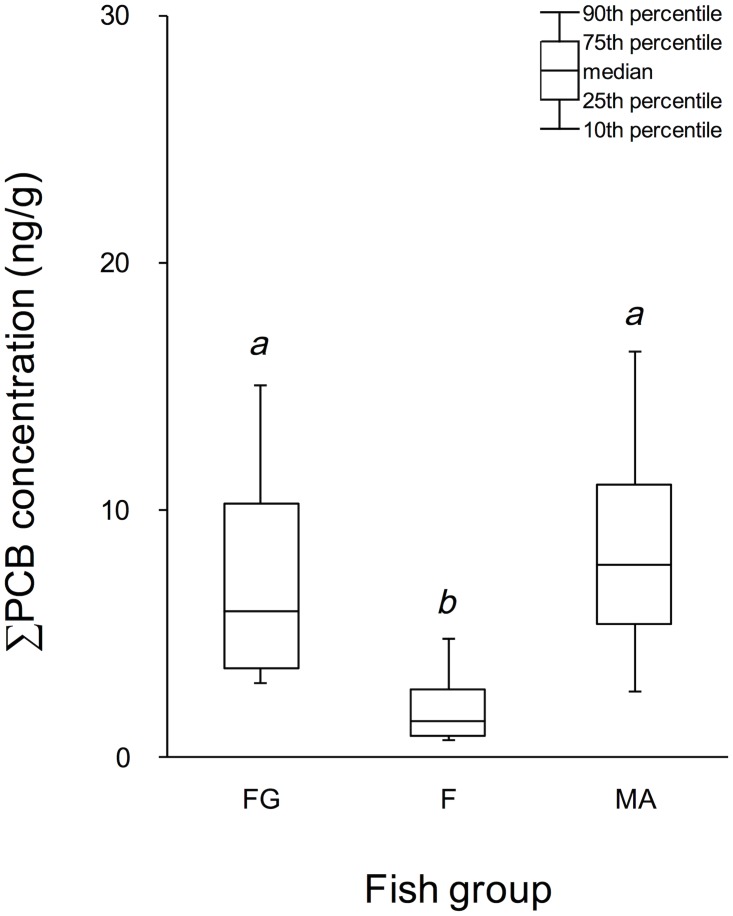
Box plots of the ∑PCB concentrations (ng/g) in Fat Greenling (FG), Flounder (F), and Mackerel (MA). Significantly different (*p* = 0.05) results are distinguished by indices *a* and *b*.

Fig 2 presents box plots of the ∑PCB concentrations in the three fish groups. The ranges of the ∑PCB concentrations between the 25^th^ and 75^th^ percentiles were very similar in fat greenling (3.6 g/g and 10 ng/g, respectively) and mackerel (5.4 ng/g and 11 ng/g, respectively). According to reports by the Ministry of the Environment of Japan (MOE) [16–20], the ∑PCB concentrations in fat greenling around the northern areas of Japan (including the investigated areas in the present study) ranged from 1.6 to 16 ng/g (*n* = 12) before the Great East Japan Earthquake in 2010 and 2011, and from 5.0 to 16 ng/g (*n* = 8) after the event. Most of the present ∑PCB levels in fat greenling were within the MOE-reported concentrations after the event, and there was no substantial difference in the ∑PCB concentrations between the MOE reports and the present results. The effect of the tsunami on the PCB contamination of marine fish was difficult to evaluate because few data were collected prior to the event in the present study areas. As shown in Fig 2, the ranges of the ∑PCB concentrations between the 25^th^ and 75^th^ percentile values were lower in the flounder samples (0.83 ng/g and 2.7 ng/g, respectively) than in the fat greenling (3.6 g/g and 10 ng/g, respectively) and mackerel (5.4 ng/g and 11 ng/g, respectively) samples. In an investigation in 2002, the ∑PCB concentrations in four flounder samples ranged from 0.92 to 19 ng/g, although but the investigation area differed from the present study area [21]. Other types of marine fish yielded higher values; sea eel contained 46–280 ng/g ∑PCB (*n* = 4), whereas flathead mullet contained 8.3–13 ng/g (*n* = 4). The 2002 report also calculated the ranges of the ∑PCB concentrations per fat weight: flounder, 150–5,900 ng/g; sea eel, 490–1,600 ng/g; flathead mullet, 1,200–1,700 ng/g [21]. These results suggest that ∑PCB is probably concentrated in the fatty tissues, as reported previously [21, 22].

### Analysis of chlorinated congeners as percentages of the ∑PCB

**Fig 3:**
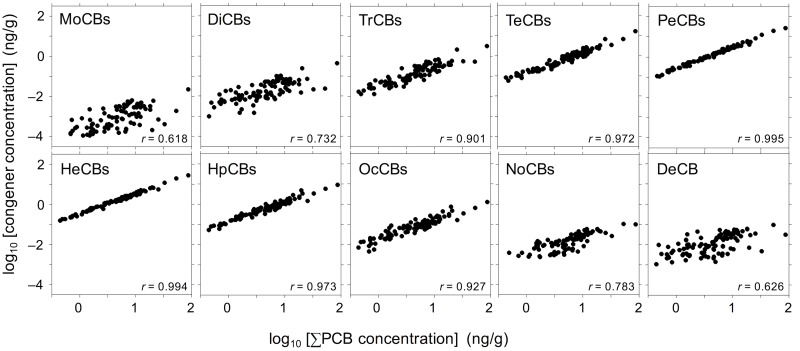
Log–log plots of chlorinated congener concentration versus ∑PCB concentration (ng/g). The statistical significance of all regressions is *α* < 0.005.

**Fig 4:**
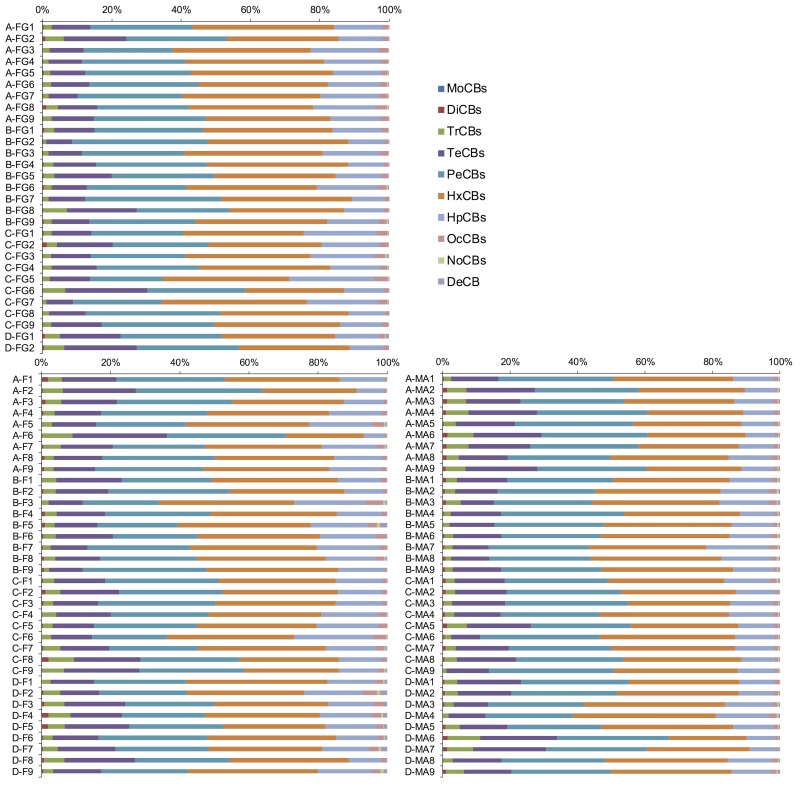
The concentrations of each chlorinated congener expressed as a percentage of the ∑PCB concentration in Fat Greenling (FG), Flounder (F), and Mackerel (MA). A–D in the sample codes represent the areas from which the samples were obtained.

We further determined the concentrations of the individual chlorinated congeners (S4 Table). Fig 3 shows graphs of the chlorinated congener concentrations plotted against the ∑PCB concentration on a log–log scale. In each case, the chlorinated congener and ∑PCB concentrations were positively correlated (the correlation coefficients are given at the bottom right of each panel in Fig 3). The correlation coefficients were lower for MoCBs, DiCBs, NoCBs and DeCB than for other chlorinated congeners, probably because their concentrations were lower, which would increase the uncertainty in their measurements [23]. In the present investigation, the concentration ratio of each chlorinated congener to ∑PCB was approximately constant, and insensitive to variations in the ∑PCB concentration. Fig 4 shows the concentrations of the chlorinated congeners as a percentage of the ∑PCB concentration. In all samples, the dominant PCBs were tetra- to hepta-chlorinated congeners (TeCBs, PeCBs, HxCBs, and HpCBs) (combined contribution >86%). In particular, the concentration proportions of the chlorinated congeners were as follows: MoCBs, ND–0.16%; DiCBs, 0.046%–2.1%; TrCBs, 1.0%–9.7%; TeCBs, 7.4%–27%; PeCBs, 21%–39%; HxCBs, 23%–43%; HpCBs, 5.9%–25%; OcCBs; 0.66%–5.2%; NoCBs, ND–0.98%; DeCB, 0.015%–2.1%. The pattern of these proportions was quite similar among the samples, although slight compositional differences were observed in some samples such as C-FG6 and A-F6. Both of these samples contained a relatively high percentage of TeCBs; however, it was difficult to determine that these differences were due to the tsunami-induced PCB contamination. We expected that the proportions of the chlorinated congeners in fish would differ significantly between conventional samples and samples freshly contaminated with PCBs. KC 300, 400, and 500, or their mixtures, were widely used as insulation in electrical condensers and transformers in Japan [4, 24]. Especially KC 300 and 400 contain a relatively large proportion of low-chlorinated PCBs (MoCBs, DiCBs, and TrCBs) at more than 15% of ∑PCB [24]. Furthermore, it has been reported that fish metabolize PCBs more slowly than do mammals [25], implying that the proportions would change slowly. If fish were contaminated with high levels of a new contaminant source of PCBs induced by the tsunami, the proportions of the low-chlorinated PCBs in fish should have been higher than those of conventional samples. Before the earthquake, the proportion of the sum of MoCBs–TrCBs to the ∑PCB in fat greenling was reported as 0.91–6.0% [16, 17]. This proportion range is close to that obtained from fat greenling in the present study (1.2–6.9%). In addition, the patterns of PCB proportions in the present investigation resemble those reported in other studies of marine fish around Japan [26, 27]. These findings imply that marine fish obtained from markets in tsunami-stricken areas in our study were unlikely to have been newly contaminated with PCBs as a result of the tsunami. It has been reported that the major congeners in KC 400, 500 and 600 were TrCBs–PeCBs, TeCBs–HxCBs, and HxCBs–HpCBs, respectively [21, 24]. In the present study, contaminating PCBs in the fish consisted mainly of TeCBs–HpCBs, and the PCB congener patterns were similar to those in fish that may have been contaminated with PCBs whose source presumably was a mixture of KC 500 and 600 [26]. In other words, the PCBs detected in marine fish in the present investigation were most likely derived from residual KCs in the environment rather than introduced by the tsunami.

### Individual congener concentrations

In all samples, the most dominant congener was #153 (comprising 7.2%–18% of the ∑PCB concentration). Because they have high lipophilicity and persistence, the predominance of #153 and #138 in marine fish has previously been reported in Japan [26, 27] as well as in fish in Europe [28–30] although the PCB source of the contamination would be different from KC in Japan. The maximum collective concentration of six indicator PCBs (#28, #52, #101, #138, #153, and #180), which are classified as NDL-PCB and used to monitor ∑NDL-PCB levels in foods in the EU [31], was 28 ng/g in a mackerel sample (Table 2). This value is below the EU regulation limit (75 ng/g for ocean fish) [32]; moreover, the concentration of #52 might have been overestimated because it was incompletely separated from #69 on the chromatogram obtained in the analysis. The sum of these six indicator PCB concentrations, relative to the ∑PCB concentration, was 29%–42% in fat greenling, 26%–40% in flounder, and 24%–36% in mackerel. These proportions do not differ from those in the investigated fish groups in the present study.

**Table 2:** Statistical data on the concentrations (ng/g) of ∑PCB, ∑NDL-PCB, and the summed concentrations of six and seven PCB indicators in fat greenling, flounder, and mackerel.

		Concentration (ng/g)				
Fish group		Mean	SD	Maximum	Median	Minimum
fat greenling (*n* = 29)						
	∑PCBs^1)^	8.1^*a*^	6.8	33	5.9	1.7
	∑NDL-PCB^1)^	7.1^*a*^	5.9	27	5.2	1.4
	∑6 indicators^1^^,^^2)^	2.8^*a*^	2.4	11	2.0	0.57
	∑7 indicators^1^^,^^3)^	3.4^*a*^	2.9	15	2.5	0.72
flounder (*n* = 36)						
	∑PCBs^1)^	2.8^*b*^	4.4	25	1.5	0.44
	∑NDL-PCB^1)^	2.5^*b*^	4.1	23	1.4	0.41
	∑6 indicators^1^^,^^2)^	0.86^*b*^	1.3	7.1	0.50	0.16
	∑7 indicators^1^^,^^3)^	1.0^*b*^	1.5	8.4	0.58	0.18
mackerel (*n* = 36)						
	∑PCBs^1)^	11^*a*^	15	86	7.8	1.6
	∑NDL-PCB^1)^	10^*a*^	14	79	7.2	1.4
	∑6 indicators^1^^,^^2)^	3.7^*a*^	5.0	28	2.3	0.54
	∑7 indicators^1^^,^^3)^	4.4^*a*^	6.0	33	2.7	0.64

^1)^ Significantly different (*p* = 0.05) results are distinguished by indices *a* and *b*.

^2)^ #28, #52, #101, #138, #153, and #180

^3)^ #28, #52, #101, #118, #138, #153, and #180

**Fig 5:**
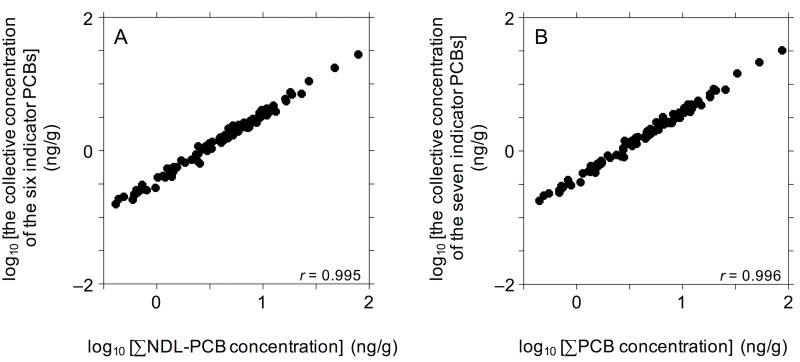
Log–log plots of the summed concentrations of (A) six PCB indicators (#28, #52, #101, #138, #153, and #180) versus the ∑NDL-PCB concentration and (B) seven PCB indicators (the previous six indicators plus #118) versus the ∑PCB concentration. All concentrations are in ng/g. The statistical significance of all regressions is *α* < 0.005.

Significant positive correlations (*r* = 0.995) were observed between the ∑NDL-PCB concentrations and the collective concentrations of the six indicator PCBs (Fig 5A). The average concentration proportion of the sum of the indicator PCBs to ∑NDL-PCB was 37% (range 26%–48%), although the European Food Safety Authority noted that these six indicators collectively account for approximately 50% of the ∑NDL-PCB in food [31]. We also calculated the collective concentration of seven indicators (the six indicators plus #118) (Table 2). In all samples, #118 was the most abundant DL-PCB, contributing 3.4–13% to the ∑PCB concentration. As observed in the six-indicator analysis, the collective concentration of the seven indicators was significantly correlated with the ∑PCB concentration (*r* = 0.996; see Fig 5B). The average concentration proportion of the sum of seven indicators to ∑PCB was 39% (range 29%–50%). Thus, we consider that the ∑PCB concentration can represent the net concentration of the specified congeners, and vice versa.

## Conclusions

In the present investigation of PCB concentrations in 101 marine fish obtained from tsunami-stricken areas, no sample was contaminated with PCB levels above the provisional regulatory limit in Japan or the regulatory limit in the EU. In the congener analysis, TeCBs, PeCBs, HxCBs and HpCBs dominated in all samples (comprising over 86% of the ∑PCB). In almost all samples, the proportion of chlorinated congeners was similar to the contamination patterns in previous reports, although slight compositional differences were observed in some samples. The average concentration proportion of the sum of six PCB indicators to the ∑NDL-PCB was 37%. Throughout the present investigation, there appeared to be no regional characteristic of the PCB contamination. Thus, any additional relationships between these compositional differences and the effects of tsunami-induced contamination are difficult to infer. To obtain more detailed information, further investigations are required using improved experimental designs such as including fish from areas not affected by the tsunami, increasing the number of fish, and introducing multivariate analyses.

## Supporting information

S1 Table Monitoring ions for the HRGC/HRMS in the present study. (XLSX)

S2 Table Recovery and precision of the recovery test (*n* = 5) for PCB congeners. (XLSX)

S3 Table The limits of LOD and LOQ concentrations for each congener in the present study. (XLSX)

S4 Table The concentrations (ng/g) of chlorinated congeners, ∑PCB, and the summed concentrations of six and seven PCB indicators in 101 fish samples. FG; fat greenling, F; flounder, MA; mackerel.
(XLSX)

S1 Text Extraction and clean-up procedures. (DOCX)
