# Supplementary material for: Determination of polychlorinated biphenyls in marine fish obtained from tsunami-stricken areas of Japan
Source: PLoS One. 2017 Apr 4;12(4):e0174961. doi: 10.1371/journal.pone.0174961 (PMC5380342; doi:10.1371/journal.pone.0174961)
Supplement: S1 Text — (DOCX) [file pone.0174961.s005.docx]

S1 Text. Extraction and clean-up procedures

The homogenized sample (20.0 g) spiked with ^13^C_12_-labeled internal standards was digested in 1M KOH–ethanol (100 mL) for 16 hours at room temperature with agitation. The digested sample was then extracted 3 times with *n*-hexane (100, 70, and 70 mL), then washed twice with 2% NaCl solution (100 mL). The extract was treated with concentrated sulfuric acid (15 mL) and the aqueous layer was removed after shaking. This step was repeated until the *n*-hexane layer became colorless. After drying the *n*-hexane layer with anhydrous sodium sulfate, the extract was cleaned on a multilayer silica gel column, which was conditioned by *n*-hexane (100 mL) before use, and eluted by *n*-hexane (50 mL). The eluate was concentrated with an evaporator. Next, the concentrated eluate was passed through an alumina column, which had been pre-conditioned by *n*-hexane (200 mL). The eluate (100 mL *n*-hexane) was concentrated with an evaporator and spiked with ^13^C_12_-labeled recovery standards. The sample solvent was gently concentrated to 100 μL under nitrogen gas.
